# Commentary to: a cross-validation-based approach for delimiting reliable home range estimates

**DOI:** 10.1186/s40462-018-0128-2

**Published:** 2018-07-12

**Authors:** Eric R. Dougherty, Perry de Valpine, Colin J. Carlson, Jason K. Blackburn, Wayne M. Getz

**Affiliations:** 1 Environmental Science, Policy & Management, University of California, Berkeley, USA; 2 National Socio-Environmental Synthesis Center, University of Maryland, Annapolis, USA; 3 Department of Biology, Georgetown University, Washington, DC, USA; 4 Spatial Epidemiology and Ecology Research Laboratory, Department of Geography, University of Florida, Gainesville, USA; 5 Emerging Pathogens Institute, University of Florida, Gainesville, USA; 6 School of Mathematical Sciences, University of KwaZulu-Natal, Durban, South Africa

**Keywords:** Time local convex hulls, T-LoCoH, Home range, Visitation, Duration, Cross-validation, Etosha national park

## Abstract

**Background:**

Continued exploration of the performance of the recently proposed cross-validation-based approach for delimiting home ranges using the Time Local Convex Hull (T-LoCoH) method has revealed a number of issues with the original formulation.

**Main text:**

Here we replace the ad hoc cross-validation score with a new formulation based on the total log probability of out-of-sample predictions. To obtain these probabilities, we interpret the normalized LoCoH hulls as a probability density. The application of the approach described here results in optimal parameter sets that differ dramatically from those selected using the original formulation. The derived metrics of home range size, mean revisitation rate, and mean duration of visit are also altered using the corrected formulation.

**Conclusion:**

Despite these differences, we encourage the use of the cross-validation-based approach, as it provides a unifying framework governed by the statistical properties of the home ranges rather than subjective selections by the user.

**Electronic supplementary material:**

The online version of this article (10.1186/s40462-018-0128-2) contains supplementary material, which is available to authorized users.

## Background

Continued exploration of the the cross-validation-based approach proposed in [1] has revealed a number of issues with the original formulation of the optimization equation. This original formulation was ad hoc in its combination of two statistical approaches (cross-validation and information criteria), and the result was a metric without a clear basis in statistical theory. As such, we strongly recommend that users rely upon the method described here as opposed to one set forth in the original publication. In particular, the shortcomings can be summarized as follows:

1. Both cross-validation and information criterion approaches aim to avoid over-fitting. In the case of cross-validation, one attempts to estimate out-of-sample prediction error, so the score used should be a measure of prediction errors of the held-out points. If the model uses *k* too small or *s* too large, it is likely to overfit the training data and will predict the testing data poorly. On the other hand, if the model uses *k* too large or *s* too small, it will underfit the training data by missing the real variations in space use. Thus, cross-validation naturally penalizes model complexity because excessive complexity (small *k*) results in poor predictions. Information criteria approaches include a penalty term that increases with model complexity as measured by larger numbers of parameters. Using such an information criterion as a cross-validation score is not necessary since cross-validation should naturally penalize excessive model complexity.
2. The formulation of the information criterion score did not follow the rules of probability because probabilities of out-of-sample predictions were not properly normalized, and multiple probabilities were combined by summation. In this sense, it lacked a firm connection to the statistical theory underlying information criteria approaches.

Here we propose an alternative formulation in which we interpret a normalized version of LoCoH hulls as an estimated probability surface and recast the cross-validation score as the total log probability of out-of-sample predictions, a common choice in cross-validation schemes. The approach, explained in detail below, results in more appropriate behavior, but also has the effect of significantly altering the optimal parameter values selected by the algorithm. Thus, in addition to presenting the new cross-validation equation, we include tables and figures with the newly selected parameter values and newly calculated derived metric values (home range area, mean duration, and mean visitation rates). Finally, we offer an alternative R script that searches a much broader parameter space in a more efficient manner (Additional file 1).

### Updated Cross-Validation Approach

**Fig. 1:**
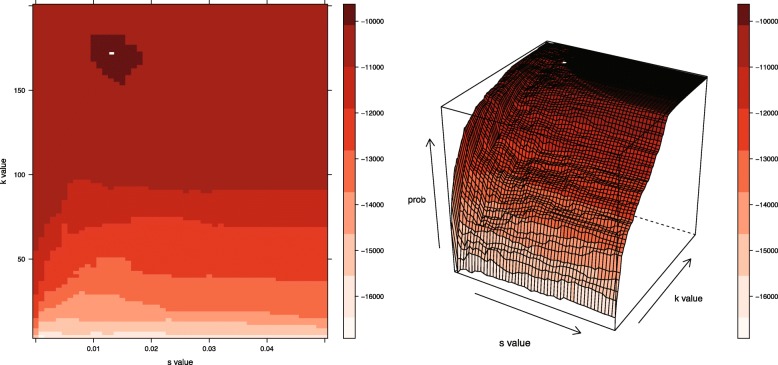
Conceptual Figure of Grid-based Search. A cross-validation surface is generated as the algorithm searches over a grid of alternative *s* and *k* values for each individual movement path. The increments of the grid can be chosen by the user. The peak in the surface indicates that the home range associated with the particular parameter set offers the highest probability for the test points. Here, the white boxes denote the maximum probability value, and thereby, the optimal parameter set

Using the training/testing split as described in the original presentation of the algorithm, a grid-based exploration of parameter space was conducted (Fig. 1), whereby each of the training/testing datasets (*i*={1,...,*n*}) was analyzed at every combination of *k* and *s* values on the grid. This analysis entailed the creation of local convex hulls with *k* nearest neighbors and a scaling factor of *s*. In all subsequent analyses, we assume that the scaling of time follows a linear formulation; however, when movement patterns more closely exemplify diffusion dynamics, an alternative equation for the TSD may be more appropriate [2]. The test points (*j*={1,...,*m*}) were then laid upon the resulting hulls.

We formulate the probabilities for out-of-sample points by normalizing the LoCoH surface so that the probability of an observation occurring at a particular location can be calculated. This value is obtained by dividing the number of training hulls that contain the test point location (*g*_*i*,*j*_) by the summed area of all training hulls (*A*_*i*_). Then, the log probability was calculated for each point per training hullset. To avoid log probability values of - *∞*, test points that were not contained within any hulls were assigned a probability value equal to the inverse of $A_{i}^{2}$, resulting in a substantially lower log probability than that of a test point contained in a single hull. Finally, a single value (*P*_*k*,*s*_) was assigned to each combination of *k* and *s* value by summing across all of the test points in all of the training/testing datasets:

$${P_{k,s}} = \sum_{i=1}^{n} \sum_{j=1}^{m} \log\left(\frac{g_{i,j}}{A_{i}}\right) $$

Because the probability of each test point is normalized based on the total area contained within all of the training hulls, there exists a natural penalty for high *k* values. For example, a *k* value equal to the number of training points (*k*_*max*_; regardless of the *s* value) will result in all hulls being identical and each test point overlapping all of the hulls. However, the large total area of the hullset when *k*=*k*_*max*_ will result in relatively small probability values for each test point (i.e., independent probability values equal to the inverse of the area of one of the hulls), effectively penalizing the parameter set containing *k*_*max*_. The underlying cross-validation procedure could very easily be extended for the optimization of the the adaptive parameter in the *a*-method (as opposed to the *k*-method) because of its scaling with the total area of the hullset.

### Results

The optimal parameter values selected using the corrected cross-validation method are substantially different from those selected in the original publication (Table 1). However, because the original formulation was not supported by cohesive statistical theory, we will discuss these new results only in reference to the guideline-based parameter values rather than comparing them to the results emerging from the published algorithm. The mean *s* value selected using the algorithm for springbok was 0.02 (SE = 0.008) and for zebra was 0.0012 (SE = 0.0005). The mean *s* value selected using the guidelines for springbok was 0.005 (SE = 0.002) and 0.017 (SE = 0.002) for zebra. Thus, the *s* values selected by the algorithm and the guidelines were not significantly different for springbok (*p*=0.10), but were for zebra (*p*<0.001). In the case of the *k* values, the optimal values selected using the algorithm were significantly higher than those resulting from the guidelines. The mean *k* value selected using the algorithm for springbok was 225.5 (SE = 66.83) whereas the mean using the guidelines was 22.5 (SE = 1.71; *p*=0.003). The same trend was observed in zebra where the mean *k* value based on the algorithm was 347.2 (SE = 54.36), whereas the mean from the guidelines was 20 (SE = 1.58; *p*=0.004).

**Table 1:** Parameter values for analysis

ID	Species	Sample	*s* (Algo)	*k* (Algo)	*s* (Guide)	*k* Range
		Points				(Guide)
AG063	Zebra	2111	**0.003**	**355**	0.023125	20-25
AG252	Zebra	3601	**0.001**	**485**	0.0140625	20-25
AG253	Zebra	3601	**0**	**156**	0.0140625	25-30
AG255	Zebra	3601	**0.001**	**405**	0.0184375	20-25
AG256	Zebra	3601	**0.001**	**335**	0.0171875	15-20
AG205	Springbok	2887	**0.05**	**182**	0.003125	25-30
AG206	Springbok	3601	**0.023**	**187**	0.00875	25-30
AG207	Springbok	3601	**0.036**	**155**	0.01140625	20-25
AG209	Springbok	2887	**0.013**	**171**	0.002421875	25-30
AG214	Springbok	2887	**0.001**	**104**	0.00265625	15-20
AG215	Springbok	2883	**0**	**554**	0.00328125	25-30

The *s* and *k* values selected using the algorithm and the guidelines in the T-LoCoH documentation. A range of *k* values were used for the Guide due to the subjective nature of parameter selection

The significantly higher *k* values emerging from the algorithm gave rise to significantly larger home ranges in both species (Table 2). In springbok, the mean home range size was 265.41 km^2^ (SE = 76.23 km^2^) using the high end of the guideline based range, and 401.64 km^2^ (SE = 127.56 km^2^) using the algorithm (*p*=0.05). In zebra, the mean home range was 694.43 km^2^ (SE = 80.81 km^2^) using the guidelines and 1081.29 km^2^ (SE = 162.17 km^2^) when the algorithm was applied (*p*=0.01). When the derived metrics were considered, however, the substantial differences in *k* values did not always result in significantly different duration (Table 3) and visitation rates (Table 4). Though the duration rates in zebra derived from the algorithm were, indeed, significantly higher than those derived using the high value from the range based on the guidelines (*p*=0.05), this was not the case for springbok (*p*=0.08). Similarly, the visitation rates emerging from the parameter sets selected by the algorithm were not significantly different from those derived based on the guidelines in either species (*p*=0.33 in springbok and *p*=0.15 in zebra).

**Table 2:** Home range areas (in square kilometers)

ID	HR Area (Algo)	HR Area (Guide Low)	HR Area (Guide High)
AG063	**1093**	571	603
AG252	**1486**	913	958
AG253	**593**	501	513
AG255	**871**	579	600
AG256	**1363**	740	798
AG205	**370**	256	268
AG206	**973**	558	588
AG207	**430**	299	318
AG209	**347**	207	216
AG214	**32**	23	25
AG215	**258**	165	177

The total area of the home range obtained using the parameter sets recommended by the algorithm and by the guidelines set forth in the T-LoCoH documentation

**Table 3:** Mean duration (MNLV) values. The derived metrics obtained using the parameter sets recommended by the algorithm and by the guidelines set forth in the T-LoCoH documentation

ID	MNLV (Algo)	MNLV (Guide Low)	MNLV (Guide High)
AG063	**48.9**	10.0	11.3
AG252	**77.3**	10.4	11.7
AG253	**2.6**	10.7	12.5
AG255	**75.1**	9.5	10.3
AG256	**42.0**	8.0	9.7
AG205	**92.6**	24.4	27.1
AG206	**80.8**	14.3	16.4
AG207	**67.9**	12.3	14.5
AG209	**78.9**	23.4	26.0
AG214	**24.7**	16.5	19.4
AG215	**2.6**	37.9	42.6

**Table 4:** Mean visitation (NSV) values

ID	NSV (Algo)	NSV (Guide Low)	NSV(Guide High)
AG063	**13.8**	5.8	6.6
AG252	**9.1**	5.6	6.3
AG253	**61.5**	15.0	16.0
AG255	**19.7**	8.1	9.5
AG256	**14.0**	7.4	8.6
AG205	**7.1**	4.2	4.5
AG206	**8.2**	6.5	6.9
AG207	**17.8**	14.9	15.7
AG209	**5.7**	3.6	3.8
AG214	**20.2**	14.6	16.3
AG215	**218.1**	6.6	6.8

The derived metrics obtained using the parameter sets recommended by the algorithm and by the guidelines set forth in the T-LoCoH documentation

## Conclusion

**Fig. 2:**
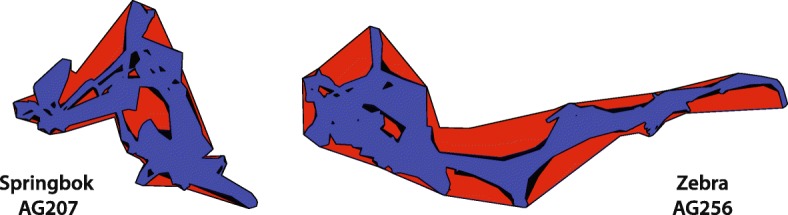
Comparison of Resulting Home Ranges. An illustration of two sets of home ranges that result from the parameter sets chosen by the algorithm (red), the low range of the guide (blue), and the high range of the guide (black). The home range set on the left is based on the sample points from the springbok AG207, and the largest home range covers 429.81 *km*^2^. The home range set on the right is based on the GPS fixes from zebra AG256, and the largest home range covers 1363.21 *km*^2^

The results presented here indicate that the effect of selecting parameters using the algorithm rather than the guidelines will be highly contingent upon the focus of the research question. Where home range delineation is the goal, the results are likely to differ significantly (Fig. 2). In the case of epidemiological questions, however, the effects will be somewhat less predictable, and in certain cases, similar conclusions might be drawn irrespective of the approach used for selecting optimal parameters. If an element of the analysis involves comparisons across individuals or species, however, the cross-validation-based approach provides a unifying framework governed by statistical properties of the home ranges rather than subjective selections by the user.

**Fig. 3:**
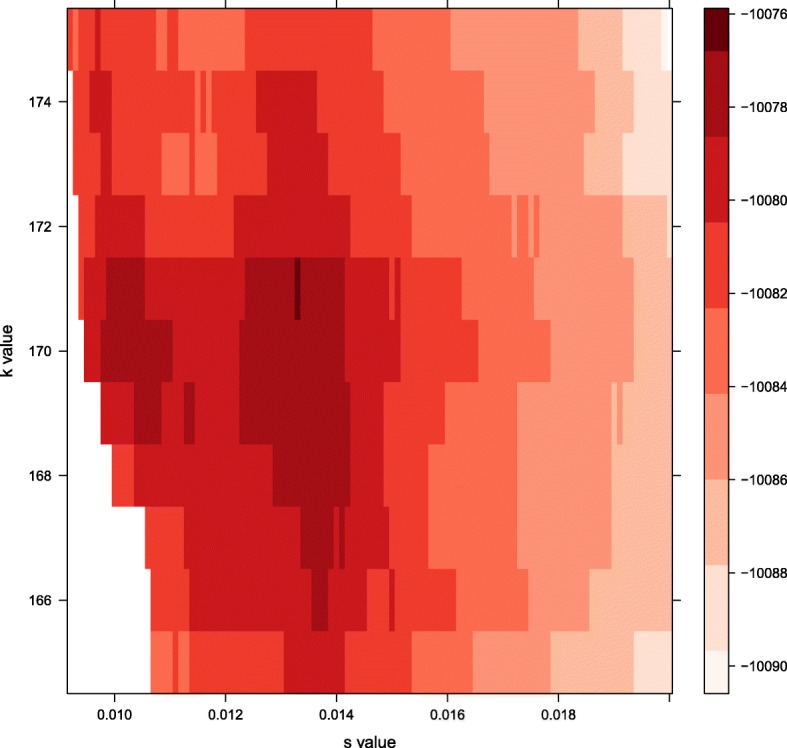
High Resolution Cross-Validation Surface. A high resolution depiction of a portion of the optimal parameter space traversed during the final stage of the efficient search algorithm. All parameter sets with log probability values above -10090 are shown, with darker shading indicating higher probability. In this particular application, the search is performed over smaller intervals of *s* (0.0001 rather than 0.001), and the optimal parameter set (*k*=171 and *s*=0.0133) is similar to the parameter set selected at the coarser scale

## Additional file

Additional file 1 A new R script for a more efficient grid-based search (Fig. 3) can be found at: https://github.com/doughertyeric/Updated_T-LoCoH_Algorithm. As currently parameterized, the grid-based search algorithm covers *s* values from 0 to 0.05 and *k* values between 4 and 800. The algorithm searches across the broadest set of *k* values in intervals of 20 and *s* values in intervals of 0.01. Upon identifying a peak in the probability surface, the algorithm selects a range of 40 *k* values around the peak and refines the search there in *k* value increments of 5. Finally, another range of 10 possible *k* values is selected and the finest scale grid-search is conducted in intervals of 1 and *s* value intervals of 0.001 before selecting the optimal parameter set. (R 11 kb)
